# #IchbinHannah and the fight for permanent jobs for postdocs: How a fictitious postdoc (almost) triggered a fundamental reform of German academia

**DOI:** 10.15252/embr.202254623

**Published:** 2022-01-31

**Authors:** Ulrich Dirnagl

**Affiliations:** ^1^ Department of Experimental Neurology Charité ‐ Universitätsmedizin Berlin Berlin Germany; ^2^ QUEST Center for Responsible Research Berlin Institute of Health Berlin Germany

**Keywords:** Careers, Science Policy & Publishing

## Abstract

Regarding postdocs as disposable labour with limited contracts is damaging for science. Universities need to offer them better career perspectives.

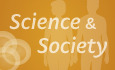

In many academic systems, permanent positions for scientists (“tenure”) are a rare exception. In Germany, 90% of the researchers employed in academia work on temporary contracts, often with less than a year’s duration. Most of the workforce on short‐term contracts are early‐career researchers (ECRs): PhD students, postdocs, or principal investigators aspiring to beome tenured professors. Given the short‐term perspectives and uncertain contract renewals, and because only a small fraction of the ECRs will eventually get a tenured position, planning the future is difficult or even impossible for them. This creates a toxic environment of hypercompetition, perverse incentives, and steep hierarchies underpinning this system, which discourages many highly competent and motivated young scientists who eventually leave in frustration. In the life sciences in particular, decisions about hiring or promotions are often based on indicators such as journal impact factor or the amount of third‐party funding. Such metrics purport to objectively quantify research quality and innovation, but instead, they foster a culture of questionable research practices, selective or non‐reporting, exaggerating the interpretation of results, and an emphasis on quantity over quality. Much has been written about this situation (Alberts *et al*, 2014), and there is a broad consensus among researchers, research administrators, funders, and learned societies on the need to reform the academic system.

Given the short‐term perspectives and uncertain contract renewals, and because only a small fraction of the ECRs will eventually get a tenured position, planning the future is difficult or even impossible for them.

## The 2007 act on temporary scientific contracts

In 2007, Germany initiated tentative first steps toward such a reform by the Act on Temporary Scientific Contracts (“Wissenschaftszeitvertragsgesetz”; https://www.bgbl.de/xaver/bgbl/start.xav?startbk=Bundesanzeiger_BGBl&jumpTo=bgbl107s0506.pdf#__bgbl__%2F%2F*%5B%40attr_id%3D%27bgbl107s0506.pdf%27%5D__1640687622967). The law limits the employment of scientists on fixed‐term contracts to 6 years after their doctorate with another qualification phase of 6 years (9 years in medicine) on the way to a professorship. Thereafter, further employment in academia can only be a permanent contract. The idea behind the law was to limit temporary contracts and encourage universities to offer researchers tenure after a period of up to 12 years. However, this was not accompanied by an increase in permanent positions at German universities. On the contrary, large national funding programs such as the “Excellence Initiative” (https://www.dfg.de/en/research_funding/programmes/excellence_initiative/index.html) have since massively increased the pool of PhD students and fixed‐term postdoc positions, further fueling the competition for limited tenured positions. The law has thus failed to achieve its intended effect, and even intensified the “triage” of well‐trained and established scientists.

These developments have fueled increasing frustration and disillusionment among Germany's ECRs. In the summer of 2021, in an ill‐fated attempt to defend the intentions of the “Wissenschaftszeitvertragsgesetz”, the German Science Ministry (BMBF) posted an animated video on its website. A fictitious cartoon character called “Hanna” introduced herself as a postdoc and explained the details of the law and the rationale behind it. Graphically and intellectually on the level of elementary school kids, the video pointed out that the law was designed to keep academia from being “clogged” by postdocs in permanent positions. Hanna further elaborated that postdocs are part of an “academic value chain” that would only lead to innovation if a substantial portion of them were continually removed from the system to make way for fresh researchers. Indeed, Hanna's view is widely held in Germany, especially among professors and university administrators. However, the video's choice of words and style was so cynical and offensive that ECRs unleashed a veritable shitstorm on social media immediately after it was published. Very quickly, the ministry removed the video from its website, but it is still available on YouTube (https://www.youtube.com/watch?v=PIq5GlY4h4E).

## #IchbinHanna

Under the hashtag #IchbinHanna (I am Hanna), thousands of ECRs who had been lectured about their role and future in the German academic system unloaded their anger and frustration on Twitter. This was picked up by many media outlets, which exposed the precarious situation of many German scientists to a broader audience. The initiators of the campaign even made it into the “Faces of 2021: who shaped the higher education headlines this year” section of the *Times Higher Education*.

It was pure coincidence that, just at this time, the parliament of the state of Berlin was debating an amendment to the Berlin Higher Education Act (https://gesetze.berlin.de/perma?d=jlr‐HSchulGBE2011V27P110); in Germany, higher education is the responsibility of the federated states (“Länder”). The ruling coalition governing the state of Berlin—social democrats (SPD), Left (“Linke”), and Green (“Grüne”) parties—was impressed by the ECRs’ campaign and the media coverage and frustrated with Berlin’s universities that, for many years, had promised to increase the number of permanent academic mid‐level positions but without any results. Without consulting the universities, the Berlin government resorted to a drastic measure and added one sentence with wide‐reaching consequences: “If the research assistant already holds a doctorate and the qualification goal stated in the employment contract is a Habilitation (an idiosyncratic German academic degree which may be obtained after the PhD), a Habilitation equivalent, the acquisition of teaching experience and teaching qualification, or other achievements to acquire the ability to work as a professor according to § 100, a follow‐up commitment must be agreed”. In other words, universities must guarantee postdocs who plan to qualify for an academic position, such as a professorship, a permanent position when they hire them for the first time, potentially immediately after they received their PhD (Vogel, 2021). What exactly would count as a qualification goal, and what the criteria might be agreed upon at the time of hiring and lead to tenure, is not further specified.

## A well‐intentioned, but badly executed law?

Although the law did not become immediately effective, Berlin’s universities panicked as soon as it was announced. Sabine Kunst, the president of Humboldt University, resigned in November last year, Free University halted all postdoctoral hiring, and the Berlin University Alliance of the city’s four largest universities and Charité Medical School terminated ongoing hiring processes of junior research groups. The universities argue that implementing the law would make one cohort of students happy, but become a disaster for all PhDs thereafter, because no permanent positions would be left for them. In addition, Berlin's universities, which have recently been labeled as “excellent” in the Excellence Strategy (https://www.dfg.de/en/research_funding/excellence_strategy/index.html), would fall behind in international competition. Professors who are to be appointed to Berlin could no longer be offered staff owing to the lack of available positions. Kunst, after her resignation, put it this way: “This law is well‐intentioned, but badly executed” (https://www.zeit.de/2021/45/berliner‐hochschulgesetz‐sabine‐kunst‐humboldt‐universitaet‐bildungspolitik‐wissenschaft). Without additional positions and without a transition phase, it would be impossible to implement.

But we should also question the mantra that permanent positions lead to a clogging of the system, and that tenured academics are less creative scientists.

There is, of course, some truth to this, and most of the discussions and media reports on the new law concern the unavoidable and understandable call for additional state funding to create more permanent positions. However, the discussion almost exclusively revolves around potentially precarious employment conditions for young academics and the structural underfunding of universities. But we should also question the mantra that permanent positions lead to a clogging of the system and that tenured academics are less creative scientists. I posit that the current discussion misses the opportunity to fundamentally rethink the structure of the academic system. Specifically, #IchbinHanna directly impacts on the quality, trustworthiness, and thus reproducibility of research.

## DOES tenure make researchers lazy and stifle creativity?

Paradoxically, most of those who warn that tenure may make researchers lazy are usually administrators on permanent contracts and tenured professors—who would vehemently reject any allegation that they have become sluggish and less committed or innovative because of a secure contract. In many other professions, in particular industry, tenure, after a probation phase, is the norm. The idea that job security stifles creativity is not only cynical but also based on a rather pessimistic concept of man: As if researchers, after being tenured, retire from the bench to the couch. The degree of self‐exploitation with which research is presently carried out—regardless of the type or length of contract after a very long apprenticeship and at any time of the day or night, as well as on moderate salaries—proves that motives other than convenience or profit drive academic researchers.

Is there any evidence for Hanna’s argument that job uncertainty and willingness to constantly change contracts, projects, or jobs are conducive to innovation? Quite the opposite. Chronic stress due to job insecurity does not improve creativity. Quality science needs time, expertise in complex methods, and routine. Interruptions and change of topic are rather counterproductive. “*Wanderjahre”* are certainly important for the training of scientists. By temporarily working in other laboratories, we learn new methods, come up with novel ideas, see how others work, establish networks, and so on. But there is no logical reason why or how permanent positions would prevent even extended visits to other laboratories. On the contrary, as scientists are inherently mobile, they could move from permanent position to permanent position at different locations. Their now vacant positions would just be filled by other scientists temporarily leaving their “home” laboratories, or permanently moving into other research groups or institutes. In addition, researchers also leave tenured positions because of retirement or taking jobs outside academia. In other words, scientists would simply move from “tenure” to “tenure” at different institutions.

## Tenure improves the quality of science

Job insecurity and constant pressure based on questionable indicators, however, have corrosive effects on science. Those who have little time and are gauged less by the substance of their research than by their h‐index may engage in less team science, conduct less transparent research, and will also be more tempted to take “shortcuts” to secure their next contract. Such shortcuts may include selective use of data, HARKING—undisclosed retrofitting of hypotheses based on results—questionable statistical methods, small studies with too few subjects, and many other practices that degrade the internal validity of research. Thus, the current employment and incentive structure may be one reason for the disappointing translatability of study results, a lack of reproducibility, and generally an inefficiency and waste of resources in science. Permanent positions reduce the pressure on scientists and are thus a bulwark against questionable scientific practices. Moreover, long‐term and even more so permanent positions free scientists from obstructive power structures that are still prevalent in many fields. Early independence promotes creativity and initiative and prevents the appropriation of achievements by others, especially those higher up in the hierarchy.

In the context of this discussion, we should also keep in mind that permanent academic positions are needed to actively support scientists in making their research more trustworthy, transparent, and useful. For example, research data management, including FAIR (findable, accessible, interoperable, reusable) sharing of data, requires specialized skills. Many scientists are willing to share their data but lack resources and the necessary know‐how. So‐called data stewards, specially trained and with previous experience in research but no longer wanting to pursue their own projects, are the solution. Another example concerns core facilities, which provide methodological competence at the highest level by staff scientists. These may be involved in research projects but are not responsible for them. Scientists in such supportive permanent positions help to reduce the inherent tension between the quality and pace of research.

Those who follow Hanna's argument that job turnover promotes innovation have succumbed to another questionable mantra: that innovation is the primary driver of science. This idea is related to the imperative to produce spectacular results that can make it into journals such as *Science*, *Nature* or *Cell*, or at least into the newspaper, thus propelling scientists to fame and glory. While this may be the mechanism by which academic careers are made, it does not map well onto how science progresses. Science is predominantly “normal” (Kuhn, 1962). Without normal science, which advances knowledge in small increments, there can be no “big” innovations or even paradigm shifts. Normal science, however, must produce robust results to be useful. Despite a number of attempts to engineer and make scientific innovation plannable (Azoulay *et al*, 2018), true innovation is exceedingly rare, unpredictable, and often the result of chance. Normal science needs time and does not work under pressure. Permanent positions support normal science.

Permanent positions reduce the pressure on scientists and are thus a bulwark against questionable scientific practices.

## Evaluation criteria for postdoc tenure

As many arguments favor early tenure options for ECRs, the pressure to change hiring practices and hierarchies in academia has been increasing during the past two decades in many countries. In Germany, the so‐called “junior professorship” was introduced in 2002 in an attempt to front‐load the decision for tenure on the professor track. However, the number of junior professorships offered has remained limited. Those who want to stay in academia, but not at the professor level, are not eligible. The new Berlin law aims at creating such an academic mid‐level career track. It does so by stipulating that postdocs must be offered the prospect of a permanent position, conditional on fulfilling certain criteria, which need to be specified at their first hiring. But which criteria would be appropriate and applicable for evaluating postdocs hoping to stay in academia, but without the ambition to become professors? As mentioned above, third‐party funding, h‐index, and journal impact factor are unsuitable. In addition, such metrics would not be appropriate for ECRs for practical reasons—after all, they have had little opportunity to score in these indicators. With ECRs, the focus must be primarily on qualitative criteria. How competent or transparent is their research? Does he or she use dissemination formats beyond classical peer‐reviewed articles, such as preregistrations or preprints? Are methods and results of high‐quality and shared FAIR, or are NULL and negative results published? Which methodological contributions are made, which impact did the research have on the discourse of the scientific community? Besides short written narratives, invitations to lectures, awards, and statements of peers are more suitable indicators.

## Front‐loading career path decisions

When front‐loading important career decisions, such as commitment to tenure track, the preparation for available academic career paths or other alternatives—in industry, public service or publishing—must begin much earlier than today and must be done better and more systematically. ECRs need to know about alternative career paths inside and outside academia. For most ECRs, current career paths in academia must appear like labyrinths, with hardly any signposts. Decisions are often made in an uninformed and rather random fashion. The role of individual luck—or alternatively bad luck—is massively underestimated: A PhD student in the right laboratory at the right time with a first authorship in a major journal is considered successful and a “high potential”. Conversely, an equally able and enthusiastic PhD student but badly mentored or charged with a floundering project might end up being labeled as probably unfit for a career in science. This is just another reason to keep scientists in the system in relative independence until they had time to prove their aptitude.

It makes little sense for societies to waste resources on training scientists for such a long time, just to let them go and work in completely different areas. In the current system, researchers must aim for a professorship to stay in academia. But only few of those are available, and indeed, many scientists are neither fit for nor interested in all the administrative duties and the necessary power play that come with running large research groups or institutes. They are eager to do research, train other scientists, or help to administer research in the laboratory, but not to sit in committees or on panels. Of course, exit strategies from academia are needed. But the decision to quit is ideally made before committing to the race for professorship, or working on a permanent position. When guiding and evaluating ECRs in this stratification, criteria must be transparent and accountable, and appropriate to the path being pursued, depending on mid‐level faculty, professorship, or leaving academia.

## The academic system needs a fundamental overhaul

While all this may sound plausible and desirable to many, why did the new law meet such vehement resistance from Berlin's universities? Resistance to a law that promises the implementation of a reform, which was discussed for a long time, and key elements of which were agreed upon among the stakeholders. Quite simply: Although the Berlin law makes a lot of sense, universities lack the financial resources to implement it and the academic system in Germany would not allow a reform of hiring of postdocs without a drastic overhaul. At present, as in many other countries, a large proportion of academic research funding in Germany is provided through project funding, which pays for PhD students and postdocs. Also, projects are, by their nature, temporary with no or little prospect of permanence.

Can a project‐oriented funding system be reformed without overhauling the organization of academic science? A straightforward option could be using a fraction of the project money for funding permanent positions. Most countries, including Germany, already work with such “overheads”. German universities receive an overhead of 20% from major funders, such as the Deutsche Forschungsgemeinschaft, DFG, and the Science Ministry, BMBF. This is, of course, far too little. In addition, universities spend the overhead on administration, infrastructure, maintenance, and so on but never on researchers. Most private funders in Germany do not pay any overhead at all. The acquisition of third‐party funding by their researchers therefore puts universities in dire financial straits, because overhead costs are not properly covered. Paradoxically, the more successful a university in attracting outside funding, the greater it is underfunded and the more limited are its resources to support researchers.

For starters, universities need a general increase in basic state funding; as German universities have been structurally underfunded for many years. In addition to receiving money to maintain their basic infrastructure, which is often crumbling, universities should get additional funds for permanent positions. By means of novel distribution schemes, universities could distribute personnel resources from a pool of permanent positions to externally funded projects. This would not work at the research group level, but certainly at an institutional level. As employers of their researchers, and custodians of the grant money they attract, they have all the data available to optimize the models through mathematical simulations before piloting implementations.

By means of novel distribution schemes, universities could distribute personnel resources from a pool of permanent positions to externally funded projects.

## Increasing the overhead and earmarking it for permanent positions

Of course, this would also necessitate an increase in the overheads for universities. Without an increase in overall funding from public funders, the additional money needed for this could come from either reducing the number of funded projects or by redistributing money between different funding streams. Decreasing the number of funded projects is not a good option. Success and innovation in research are not predictable, and progress mostly comes from many researchers engaged in “normal” science, and a few breakthrough projects. Another ill effect of such a constriction in project funding would be further heating up an already unhealthy competition with all its negative consequences. The solution may come from the fact that a large fraction of the German research funding goes into major programs such as the Excellence Initiative mentioned above. These have merit as they provide resources without which university research in Germany would be entirely dysfunctional. However, as such programs are made up of many small projects, they rather perpetuate the vicious circle of generating an army of PhD students and postdocs on fixed‐term contracts without career prospects. Rerouting at least part of the financial resources of these large programs into overheads for permanent positions would help to finance overdue reforms, such as the one introduced by the state of Berlin.

At the same time, politicians need to create the legal basis for such a reform in a dialog with the universities. It may help that Germany has just got a new government. According to its coalition agreement, it plans to reform the German Act on Temporary Academic Contracts (Wissenschaftszeitvertragsgesetz) mentioned above, and increase the predictability of the postdoc phase. In addition, the governing coalition pledges to foster alternative careers to the professorship track, an expansion, and continuation of the tenure track program, as well as to create permanent positions for permanent tasks. All this sounds almost too good to be true, as it promises to set the ground for an overdue reform the German academic system, while at the same time improving the conditions for young academics, increasing the quality of research, and making Germany more attractive for scientists. Perhaps even other countries whose science systems are plagued by similar structural problems might take note. Also, Hanna would be amazed to realize the tremendous impact of her little video.
